# Supplementary material for: Regional characterization of meteorological and agricultural drought in Baluchistan province, Pakistan
Source: PLoS One. 2024 Aug 19;19(8):e0307147. doi: 10.1371/journal.pone.0307147 (PMC11332948; doi:10.1371/journal.pone.0307147)
Supplement: Attachment — Submitted filename: PLOS.docx [file pone.0307147.s001.docx]

The research entitled “Regional Characterization of Meteorological and Agricultural Drought in Baluchistan Province, Pakistan” aimed to examine the spatiotemporal pattern of three competitive drought indices (RDI, SPEI, and VCI) in Balochistan province of Pakistan.

The study is important to reveal the regional climatic characteristics of largest province of Pakistan and related to its vegetative (Agricultural) phenomenon. Overall, research design is valid but substantial improvements are needed. My specific comments are:

1. Although sample stations are good representative of study area, but the methods are very simple and presents less novelty. Research design need to be enhanced adding drought frequency and trend analysis. Follow the drought run theory.
2. How VCI is calculated? Which remote sensing dataset used? The method is not properly described. Which temporal scale is used? If it is calculated using Landsat, the results might be uncertain. MODIS is more recommended for a province level study area and will provide more consistent results. In this case, VCI can be regressed with 20 years analysis (based on the dates of datasets). At what temporal scale VCI is generated? 12 months? Clarify the methodology.
3. Spatial presentation of RDI and SPEI can be added for spatial comparison with VCI at same scale.
4. A Pearson correlation matrix can be added for all indices for more clear statistical relationship between the variables. Regression scatterplot reveals a very weak relationship of VCI with SPEI. Need to be discussed, why? No need of table 8
5. Discussion part need to be more strong with more relevant reference and discussing the major causes of previous drought events.

Overall, research design of the study need to be enhanced adding more sound methods and results.
