# Supplementary material for: Regional characterization of meteorological and agricultural drought in Baluchistan province, Pakistan
Source: PLoS One. 2024 Aug 19;19(8):e0307147. doi: 10.1371/journal.pone.0307147 (PMC11332948; doi:10.1371/journal.pone.0307147)
Supplement: Attachment — Submitted filename: Response to PlosOne Comments (1).docx [file pone.0307147.s002.docx]

***Regional Characterization of Meteorological and Agricultural Drought in Baluchistan Province, Pakistan***

**Point-to-Point Response to the Reviewer’s Comments**

We are thankful to the editor and team for their timely response and efforts. We are also thankful to reviewers for their deep and valuable comments that improved the quality of the manuscript. We are submitting our point-by-point response to the comments and an updated manuscript file with highlighted **in red colour**. We are hopeful that the revised manuscript has improved to the level of satisfaction and approval. We also hope that now the manuscript meets the journal’s publication requirements. Number-wise answers to their specific comments and suggestions are as follows.

**Editor Comments:**

*Please ensure that your manuscript meets PLOS ONE's style requirements, including those for file naming. The PLOS ONE style templates can be found at*

**Authors response:** We highly appreciate your effort. We carefully checked the format and set according to the journal requirements.

*Please note that funding information should not appear in any section or other areas of your manuscript. We will only publish funding information present in the Funding Statement section of the online submission form. Please remove any funding-related text from the manuscript.*

**Authors response:** We removed the funding information from the manuscript.

*We note that the grant information you provided in the ‘Funding Information’ and ‘Financial Disclosure’ sections do not match.*

**Authors response:** We reconfirmed the provided grant information and correct it carefully.

*For studies involving third-party data, we encourage authors to share any data specific to their analyses that they can legally distribute. PLOS recognizes, however, that authors may be using third-party data they do not have the rights to share. When third-party data cannot be publicly shared, authors must provide all information necessary for interested researchers to apply to gain access to the data. (*[*https://journals.plos.org/plosone/s/data-availability#loc-acceptable-data-access-restrictions*](https://journals.plos.org/plosone/s/data-availability#loc-acceptable-data-access-restrictions)*).*

**Authors response:** No third party data involve in this research.

*PLOS requires an ORCID iD for the corresponding author in Editorial Manager on papers submitted after December 6th, 2016.*

**Authors response:** ORCID ID provided for the corresponding author.

*We note that Figure 1 and 6 in your submission contain map/satellite images which may be copyrighted.*

**Authors response:** The figure 1 and 6 is solely the authors own maps. There is no copy right issue. These are prepare under Creative Commons Attribution License (CC BY 4.0)." Authors response: The figure 1 and 7 is solely the authors own maps. The Base layers used in Figure 1 are: Digital Elevation Data which is freely available from Website 30-Meter SRTM Elevation Data Downloader (dwtkns.com). The Shape files used in preparing map for Figure 1, 2 and 7 is digitized from the Map available from Survey of Pakistan Website: <http://www.surveyofpakistan.gov.pk/Detail/MTUzYWU5ZGItNTA4NS00MDlkLWFlODctNTRkY2JmNWI0Mjg2>. In the figure 7 we have used Landsat NDVI data which have been discussed in the methodology section and properly cited the Landsat imageries.

Further online Base map has been removed to avoid any Copy right issue.

*Please ensure that you refer to Figure 1 in your text as, if accepted, production will need this reference to link the reader to the figure.*

**Authors response:** Reference to the Figure 1 provided in the text.

*We note you have included a table to which you do not refer in the text of your manuscript. Please ensure that you refer to Table 3 in your text; if accepted, production will need this reference to link the reader to the Table.*

**Authors response:** Reference to the Table 3 provided in the text.

**Reviewer #1:**

*General comments: In order to identify drought episodes in Baluchistan, a comprehensive analysis was conducted using three key indices: the Reconnaissance Drought Index (RDI), Standardized Precipitation Evapotranspiration Index (SPEI), and Vegetation Condition Index (VCI). This analysis took into account various factors such as precipitation, temperature, potential evapotranspiration (PET), and vegetation condition. The work is original, but I recommend that the following major changes be made before it is accepted for publication.:*

**Authors response:** We highly appreciate the effort of the reviewer for the detail review of the manuscript. The reviewer comments and suggestions improved the quality of the manuscript and we authros are highly indebted for your kind words.

*Why the selected models were chosen and their advantages should be expressed.*

**Authors response:** We discussed the advantages of each model in the revised introduction section and highlighted the importance of these models that why we selected these.

*My literature section is limited, sources from 2023 and 2024 should be added.*

**Authors response:** We highly appreciate the effort of the reviewer for the detail review of the manuscript. Authors added new citation and literature especially citations from 2023 and 2024.

*After its first mention, vegetation drought index (VCI) should be abbreviated.*

**Authors response:** We abbrevaited the Vegetation Drought Index (VCI) in the revised manuscript as suggested by worthy reviewer.

*In order to provide a more comprehensive literature section, it is suggested to incorporate the following paper. Comparison of meteorological indices for drought monitoring and evaluating: a case study from Euphrates basin*

**Authors response:** We highly appreciate the effort of the reviewer for the detail review of the manuscript. We cited the suggested research article in the Introduction Section.

*It is necessary to verify all equations. Equation 8 contains several missing and erroneous statements that need to be addressed. Here is an explanation of how the spatio-temporal distribution of drought was formed. To enhance the method section, it is advisable to employ the state interpolation method and provide a comprehensive explanation of the interpolation technique.*

**Authors response:** We rechecked all the equations and statements relevant to the given equations and corrected. We added IDW interpolation technique in the methodology section and prepared maps in the results.

*Please cite fallowing paper fort his aim: • Spatio-temporal analysis of meteorological and hydrological droughts in the Euphrates Basin, Turkey. • Spatial analysis of seasonal precipitation using various interpolation methods in the Euphrates basin, Turkey.*

**Authors response:** We appreciate your efforts. We added the suggested research article in citations to improve the literature section.

*In the introduction section, it is important to highlight the advantages of interpolation methods. Interpolation methods offer several benefits that make them valuable in various fields. Firstly, they allow for the estimation of values between known data points, providing a continuous representation of the data. This helps in accurately predicting unknown values and filling in gaps in the data set. Additionally, interpolation methods aid in reducing noise and smoothing out irregularities in the data, leading to a more precise and reliable analysis. Furthermore, these methods are computationally efficient and can handle large datasets efficiently. By incorporating interpolation methods into the introduction section, readers will gain a deeper understanding of the significance and usefulness of these techniques.*

**Authors response:** We enhanced the introduction and methodology sections in light of your valuable suggestions. Thank you for your time and comments.

The discussion section is not deep. It would be good to improve. Suggestions for future studies can be made and it can be suggested which other water resources problems the wavelet transform can be used to solve.

**Authors response:** We improved the discussion section as well and tried to give future research direction and mitigation strategies. Thank you for your valuable suggestions and time.

**Thank you for your valuable comment and time.**

**Reviewer #2:**

*The research entitled “Regional Characterization of Meteorological and Agricultural Drought in Baluchistan Province, Pakistan” aimed to examine the spatiotemporal pattern of three competitive drought indices (RDI, SPEI, and VCI) in Balochistan province of Pakistan.*

*The study is important to reveal the regional climatic characteristics of largest province of Pakistan and related to its vegetative (Agricultural) phenomenon. Overall, research design is valid but substantial improvements are needed. My specific comments are:.*

**Authors response:** Thank you for your thoughtful comments regarding our paper. We are pleased to hear that you consider our study importance. Your input is invaluable in improving the quality and impact of our research paper.

1. *Although sample stations are good representative of study area, but the methods are very simple and presents less novelty. Research design need to be enhanced adding drought frequency and trend analysis. Follow the drought run theory.*

**Authors response:** Thank you for your valuable feedback. We added the drought frequency and trend analysis following the droughy run theory. The figures are redesigned in R based on the output of run theory that shows the freuency and trend of each drought category with different colors. We also added frequency table for further explanation.

2. *How VCI is calculated? Which remote sensing dataset used? The method is not properly described. Which temporal scale is used? If it is calculated using Landsat, the results might be uncertain. MODIS is more recommended for a province level study area and will provide more consistent results. In this case, VCI can be regressed with 20 years analysis (based on the dates of datasets). At what temporal scale VCI is generated? 12 months? Clarify the methodology.*

**Authors response:** We improved the methodology section for VCI calculation and explained the datasets used. We calculated VCI based on Landsat NDVI datasets derived from Google Earth Engine. For an area like Baluchistan, Landsat provides more accurate results as the area remains cloud-free, and the data is available for a more extended period than MODIS. VCI is calculated from April to July, taking the average value of this period since, at this time, most of Baluchistan is covered with green vegetation. Therefore, we selected this period for VCI analysis.

3. *Spatial presentation of RDI and SPEI can be added for spatial comparison with VCI at same scale..*

**Authors response:** We appreciate your time and valuable comments. Considering the paper's requirements, we believe it is already quite lengthy, so we decided not to add further maps, as this would increase the length significantly. We hope you understand our decision to skip this valuable suggestion. We acknowledge that it would enhance the quality of the paper, but we apologize for not including it due to the paper's current length.

4. *A Pearson correlation matrix can be added for all indices for more clear statistical relationship between the variables. Regression scatterplot reveals a very weak relationship of VCI with SPEI. Need to be discussed, why?*

**Authors response:** We added the Pearson Correlation Matric in the revised manuscript and discuss the possible reason of low correlation of SPEI and RDI with VCI.

*5. Discussion part need to be more strong with more relevant reference and discussing the major causes of previous drought events. Overall, research design of the study need to be enhanced adding more sound methods and results..*

**Authors response:** Thank you for your constructive feedback. We added further discussion specifically on the causes of previous droughts in the study area. We thoroughly revised the manuscript and hopefully you will find it in more improved form.

**Thank you for your valuable comment and time.**
