# Supplementary material for: Regional characterization of meteorological and agricultural drought in Baluchistan province, Pakistan
Source: PLoS One. 2024 Aug 19;19(8):e0307147. doi: 10.1371/journal.pone.0307147 (PMC11332948; doi:10.1371/journal.pone.0307147)
Supplement: Attachment — Submitted filename: PLOSR1.docx [file pone.0307147.s003.docx]

Research: **Regional characterization of meteorological and agricultural drought in Baluchistan 1 province, Pakistan**

Although authors have made substantial changes to the manuscript and addressed the comments. Still, a few parts need to be improved

1. Introduction: provide a few more recent drought example studies from Pakistan.
2. The resolution of newly added figures is not good. Please improve the presentation of figure 3, 4, 5 and 6. Increase the legend and label size and try to concise them to take less space. Captains need to be revised. Remove the word “spatio” from these titles.
3. Present the statistical analysis with a good heading. Replace “Correlation statistical tool” with term “statistical analysis through Pearson correlation coefficient and linear regression”.
4. What about Mann-Kendall and Sen’s slope analysis? Why not performed?
5. Align all figures equally in manuscript.
