# Supplementary material for: Regional characterization of meteorological and agricultural drought in Baluchistan province, Pakistan
Source: PLoS One. 2024 Aug 19;19(8):e0307147. doi: 10.1371/journal.pone.0307147 (PMC11332948; doi:10.1371/journal.pone.0307147)
Supplement: Attachment — Submitted filename: Author Response.docx [file pone.0307147.s004.docx]

Research: **Regional characterization of meteorological and agricultural drought in Baluchistan province, Pakistan**

We revised the manuscript according to the directions of the editor and reviewers.

**Journal Requirements:**

Please review your reference list to ensure that it is complete and correct. If you have cited papers that have been retracted, please include the rationale for doing so in the manuscript text, or remove these references and replace them with relevant current references. Any changes to the reference list should be mentioned in the rebuttal letter that accompanies your revised manuscript. If you need to cite a retracted article, indicate the article’s retracted status in the References list and also include a citation and full reference for the retraction notice.

**Author Response:**

We carefully checked all the references in the list on Retraction Watch, and based on the information obtained, none of the references have been identified as retracted.

**Reviewer 1:**

Can accept Paper after the font size of the letters in Figure 3 is enlarged.

Author Response: We revised the figures and enlarged the font size.

**Reviewer 2:**

1. Introduction: provide a few more recent drought example studies from Pakistan.

Author Response: We included the latest literature and examples from Pakistan in both the Introduction and Discussion Sections.

2. The resolution of newly added figures is not good. Please improve the presentation of figure 3, 4, 5 and 6. Increase the legend and label size and try to concise them to take less space. Captains need to be revised. Remove the word “spatio” from these titles.

Author Response: We improved the presentation of the mentioned figures and increased the font size used in the figures. We condensed the figures to minimize the occupied space.

3. Present the statistical analysis with a good heading. Replace “Correlation statistical tool” with term “statistical analysis through Pearson correlation coefficient and linear regression”.

Author Response: We revised the heading as suggested by the reviewer.

4. What about Mann-Kendall and Sen’s slope analysis? Why not performed?

Author Response: The focus of this research was on drought, so we did not use any trend statistics in this research.

5. Align all figures equally in manuscript.

Author Response: We carefully checked and aligned all the figures.
